# The most fundamental and popular literature on functional near-infrared spectroscopy: a bibliometric analysis of the top 100 most cited articles

**DOI:** 10.3389/fneur.2024.1388306

**Published:** 2024-05-02

**Authors:** Jiyang Li, Yang Li, Maomao Huang, Dan Li, Tenggang Wan, Fuhua Sun, Qiu Zeng, Fangyuan Xu, Jianxiong Wang

**Affiliations:** ^1^Rehabilitation Medicine Department, The Affiliated Hospital of Southwest Medical University, Luzhou, Sichuan, China; ^2^Rehabilitation Medicine and Engineering Key Laboratory of Luzhou, Luzhou, Sichuan, China

**Keywords:** near-infrared spectroscopy, activation, cerebral-blood-flow, brain, newborn-infants, oxygenation, cortex, fMRI

## Abstract

**Background:**

Functional near infrared spectroscopy (fNIRS) has developed rapidly in recent years, and there are more and more studies on fNIRS. At present, there is no bibliometric analysis of the top 100 most cited articles on fNIRS research.

**Objective:**

To identify the top 100 most cited articles on fNIRS and analyze those most fundamental and popular articles through bibliometric research methods.

**Methods:**

The literature on fNIRS of web of science from 1990 to 2023 was searched and the top 100 most cited articles were identified by citations. Use the bibliometrix package in R studio and VOSviewer for data analysis and plotting to obtain the output characteristics and citation status of these 100 most cited articles, and analyze research trends in this field through keywords.

**Results:**

A total of 9,424 articles were retrieved from web of science since 1990. The average citation number of the 100 articles was 457.4 (range from 260 to 1,366). *Neuroimage* published the most articles (*n* = 31). Villringer, A. from Leipzig University had the largest number of top 100 papers. Harvard University (*n* = 22) conducted most cited articles. The United States, Germany, Japan, and the United Kingdom had most cited articles, respectively. The most common keywords were near-infrared spectroscopy, activation, cerebral-blood-flow, brain, newborn-infants, oxygenation, cortex, fMRI, spectroscopy. The fund sources mostly came from National Institutes of Health Unitd States (NIH) and United States Department of Health Human Services (*n* = 28).

**Conclusion:**

*Neuroimage* was the most popular journal. The top countries, institutions, and authors were the United States, Harvard University, and Villringer, A., respectively. Researchers and institutions from North America and Europe contributed the most. Near-infrared spectroscopy, activation, cerebral-blood-flow, brain, newborn-infants, oxygenation, cortex, fmri, spectroscopy, stimulation, blood-flow, light-propagation, infants, tissue comprise the future research directions and potential topic hotspots for fNIRS.

## 1 Introduction

Functional near infrared spectroscopy (fNIRS) is an emerging and powerful non-invasive brain imaging technique, and research on fNIRS has increased dramatically in recent years (1). fNIRS is derived from near infrared spectroscopy (NIR, 1000–2,500 nm). NIR is a vibration spectroscopy technique, the key application fields of it are bioanalytical research and biomedical diagnosis, such as body fluid, blood analysis, cell research, biomolecular research, etc. (2). In medicine, it can be used for diagnosis of coronary artery disease, monitoring and management of anemia, detection of thrombosis related diseases, bedside monitoring of shock patients, etc. (3–5). Biological tissue, including bone, is quite transparent to light in the 650–925 nm infrared wavelength range, hence near-infrared spectroscopy can be used to monitor activity in the cerebral cortex, which is located beneath the skull. Neural activity corresponds with changes in blood volume basing on the theory of neurovascular coupling, which provides a precise measure of brain activity. Based on the concept of NIRS, fNIRS measures changes in oxygen and deoxygenated hemoglobin in the brain by scattering near-infrared light between 600 and 900 nm, thereby conveniently reflecting neuronal activity in the brain in real time (6). In fNIRS and diffusion-correlated tomography, it is important to simulate light propagation within the human head to infer spatial sensitivity distributions (SSDS). The Chinese human head Visual model (VCH) is considered to be the most realistic model of the anatomical structure, and is mainly used in light propagation modeling (7).

In addition to fNIRS, Electroencephalogram (EEG) and functional magnetic resonance imaging (fMRI) are related techniques for functional brain imaging. NIRS measures variations in blood flow, while EEG measures changes in electrical changes in the brain. While fNIRS has greater spatial goals but is constrained by poorer temporal resolution because of slower hemodynamic responses, EEG require spatial targets but have higher transient targets (8). Even though fMRI has grown to be a mainstay of functional neuroimaging and is frequently used to describe neural activity in the brain, it also has some limitations; for example, it requires a high-field-intensity MRI system and requires subjects to be magnetically safe; and it measures blood oxygen changes indirectly rather than directly measuring neuronal activity. The ratio of oxyhemoglobin to deoxyhemoglobin is the basis for the blood-oxygen-level-dependent (BOLD) response as detected by fMRI (9). fNIRS, which can measure two hemoglobin levels independently, is safe for those who have implanted devices that are incompatible with MRI, such as pacemakers, deep brain stimulators, or vagal nerve stimulators (VNS). Furthermore, because of its technological benefits and device mobility, fNIRS is simpler to use than EEG and fMRI for immediate, real-time bedside assessment as well as continuous monitoring during activity or movement (4). Simultaneously, fNIRS can be integrated with current brain imaging methods to broaden their scope and better meet the demands of scientific and clinical research. Additionally, novel combinations like fNIRS + EEG (10, 11) or fNIRS + fMRI (12, 13) will be developed in particular when it comes to examining for diseases and increased understanding the efficacy.

As a monitoring method for neuroimaging and brain metabolism, fNIRS technology has high temporal resolution and moderate spatial resolution. Device, programming, and inspection applications for fNIRS have advanced quickly in recent years (14). Examples of these developments include downsized, customized, and fast inspection fNIRS devices (15). Furthermore, to improve our comprehension of the brain, fNIRS can be combined with neuroregulatory methods including transcranial magnetic stimulation (TMS), brain-computer interfaces (BCI), and transcranial electrical stimulation (16, 17). Consequently, over the last two decades, fNIRS has been applied much more frequently in clinical settings, particularly in the field of neurology (18). It has been used to investigate the cortical activation of a wide range of mental disorders, brain and non-brain diseases, and normal human (19, 20), including cognitive, speech, Parkinson’s disease, autism, and stroke, among others (21–25). Similar to NIRS, in recent years, low-level light/laser therapy (LLLT) technology based on effective photobioregulation is emerging as a new non-invasive treatment for stroke. Visible Chinese human (VCH) and Monte Carlo method (MCVM) have great potential to optimize LLLT treatment parameters for stroke and guide future LLLT treatment instruments for hemorrhagic stroke (26). However, more research is needed on the application of NIR spectroscopy in the medical field. In clinical aspects, the time point of NIR imaging intervention and the types of diseases applied still need to be explored. In terms of technology, the model selection of NIR spectral imaging and the artifact correction system need to be upgraded (27).

Bibliometric analysis is an effective technique for analyzing research performance and identifying influential articles in particular fields (28). It can assist researchers in determining priorities and trends in a special area (29). The results of bibliometrics could be helpful for decision-making and further study. There has been some fNIRS bibliometrics at present, which centers around a bibliometric visualization analysis of producers and keywords (30). As far as we know no citation analysis of the researches on fNIRS has been done. The citation analysis can identify the highly cited articles in this field. Articles with a high citation frequency can provide much basic useful information about the current state of research in a particular field (31). Highly cited articles reflect the research basis of this field and help researchers to understand this field quickly. In recent years, a large number of articles about fNIRS have emerged, so it is necessary to do a citation analysis to understand the research basis in this field. Hence, we conducted a qualitative and quantitative analysis to aid researchers in comprehending the study quality and trends of fNIRS, effectively classify the most popular article on fNIRS research, and serve as a reference for upcoming research in this area.

Currently, the commonly utilized bibliometric analysis software includes Citespace, VOSviewer, and the Bibliometrix package based on R language. These three tools have similar functions; for example, they can all analyze scientific research collaborations and co-citations based on co-occurrence frequencies, can analyze research trends and hot spots in a special field through keyword co-occurrence maps, and all can be used to build and visualize bibliometric maps. Compared with Citespace, VOSviewer is much simpler to operate and can meet the core bibliometric analysis needs, including analyzing overall research trends within related fields, tracking recent advancements, examining country-specific research landscapes, identifying institutional distributions, as well as highlighting significant researchers and influential literature. Moreover, it facilitates the identification of prominent academic groups along with their representatives, core technological areas, hot topics through intuitive visual representations (32–34). When the number of articles to be processed is not too large, VOSviewer can very well meet the needs of bibliometric analysis. The Bibliometrix package based on R language, which is a recently developed powerful visualization software, can automatically handle large volumes of data effectively, generate diverse scientific maps and statistical indexes and tables, such as M-index, G-index, Coupling Map, Three-Fields Plots, Word Dynamics, topic dendrogram, etc. Additional data and charts of Bibliometrix package can complement the outputs obtained from VOSviewer, thus both tools together facilitate a more comprehensive visual analysis of bibliometrics (35).

## 2 Method

We used the Web of Science core collection (WoSCC, index: Science Citation Index Expanded) as the retrieval tool on March 14, 2024. The search time range is from January 1, 1990 to December 31 2023. And we used [“functional near-infrared spectroscopy” (Topic) OR “functional near infrared spectroscopy” (Topic) OR fNIRS (Topic)] OR [(“near-infrared spectroscopy” (Topic) OR “near infrared spectroscopy” (Topic) OR NIRS (Topic)] AND [brain (Title) OR brain (Abstract) OR cerebral (Title) OR cerebral (Abstract) OR cranium (Title) OR cranium (Abstract) OR cortical (Title) OR cortical (Abstract)] as the retrieval strategy. According to the citations, if there were no relevant articles at the top 100, the next article would be enrolled, and the top 100 most cited articles were exported for further analysis. Two researchers simultaneously searched according to the above search strategy. No patient or public participation was involved in this study, so ethical approval was not required for this study.

## 3 Data collection and analysis

We extracted vital information from WoSCC such as author, institution, country/region, title, publication year, citation frequency, fund, journal name, and impact factor (IF).

Microsoft Excel 2021, VOSviewer (CWTS, Netherlands) and R software (v 4.1.0) were used for descriptive statistical analysis and generation of graphs. The R software package “Bibliometrix” was used for ranking analysis by country, institution, and author (36). VOSviewer is a free computer program widely used in the bibliometric analysis for creating and viewing bibliometric maps (37). Keyword co-occurrence maps and author, country, and institution collaboration maps are produced by using VOSviewer. In a network visualization map, different nodes represent different elements, such as countries, institutions, authors, or terms. Links between nodes represent relationships such as co-authorship, co-citation, or co-occurrence and are weighted by total link strength (TLS) (38). Co-authorship analysis measures cooperative linkages between countries or institutions. The relevance of nodes is determined based on the number of co-authored documents. Co-citation analysis measures the relationship between nodes based on the number of times the same document cites a node. As for co-occurrence analysis, the relevance of nodes is determined based on the number of records in which they occur together (39). The size of the nodes reflects the number of outputs, citations, or occurrences, and the colors indicate different clusters or average the year of occurrence (AAY) of these elements.

## 4 Results

**Figure 1:**
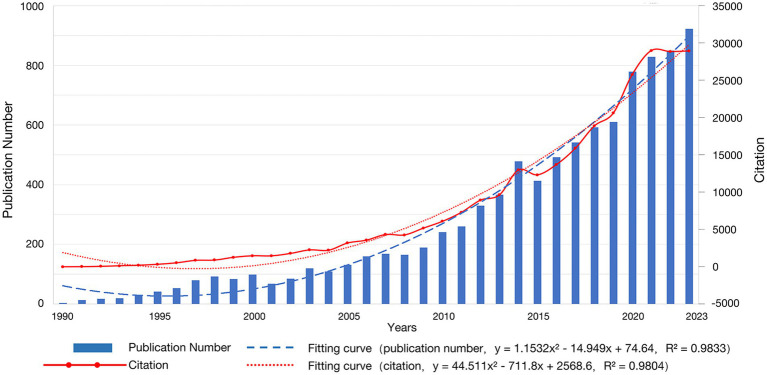
Annual publication curve.

According to our search strategy, we detected a total of 10,358 papers. After retaining only articles and reviews and filtering the articles whose language was English, a total of 9,424 articles were left. Since 1990, fNIRS research has been increasing rapidly, from 3 articles in 1990 to 922 articles in 2023 (Figure 1). Through polynomial fitting (*y* = 1.1532x^2^–14.949x + 74.64,*R*^2^ = 0.9833), it is expected that in 2024, 964 fNIRS-related articles will be published, which is shown in Figure 1. And for the citation (*y* = 44.511x^2^–711.8x + 2568.6, *R*^2^ = 0.9804), it is expected that fNIRS-related articles will be cited 32,181 times in 2024. These 9,424 articles were sorted in descending order of citation count, and the top 100 literatures were included.

### 4.1 Publications

Among the 100 publications, there are 67 articles and 33 reviews. These 100 highly cited articles mainly involve Neurosciences (*n* = 54), Radiology Nuclear Medicine Medical Imaging (*n* = 39), Neuroimaging (*n* = 32), Physiology (*n* = 14), Clinical Neurology (n = 8), Engineering Biomedical (*n* = 7), Optics (*n* = 7), Sport Sciences (*n* = 7) etc.

**Figure 2:**
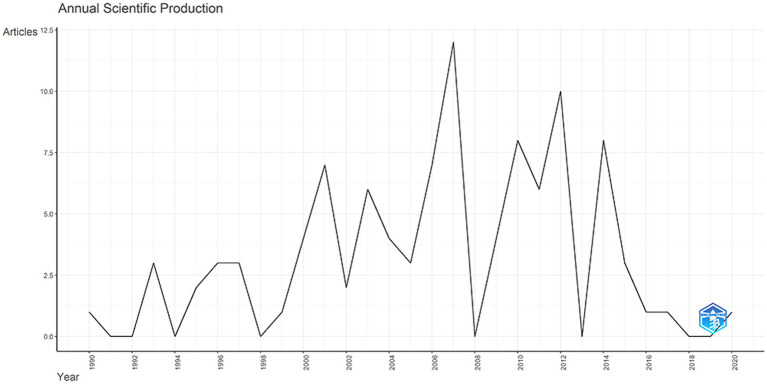
Top 100 most cited articles’ annual scientific production.

The top 100 articles were published between 1990 and 2020, with the highest number published in 2007 (*n* = 12), shown in Figure 2. As of the date of our search, the 100 articles had a total of 45,744 citations. The mean citation is 457.4 (range from 260 to 1,366), and five had more than 1,000 citations.

### 4.2 Journals

These articles have been published in 46 journals. The top 3 journals and IF are shown in Table 1. Among these top 3 journals, *Neuroimage* published the most articles (*n* = 31), followed by *Applied Optics* (*n* = 4). *Journal of Biomedical Optics*, *Journal of Cerebral Blood Flow and Metabolism*, *Pediatric Research*, *Physics in Medicine and Biology*, *Physiological Reviews*, *Stroke* (*n* = 3) tied for third. These journals accounted for 53% of the top 100 most cited articles. In terms of total citations, *Neuroimage* topped the list with 15,956 citations. Meanwhile, as for mean citation per article, *Applied Optics* took the first place with 575.0 citations per article. The IF of the top 10 journals were between 1.9 and 33.6, and their JCR zone mainly belongs to Q1, and four journals have an IF of more than 5. An article published in *Physiological Reviews*, which has the highest impact factor and citations, was *Coupling of brain activity and cerebral blood flow: basis of functional neuroimaging* was cited 502 times (40).

**Table 1:** Top 3 journals in the top-100 articles.

**Journal**	**Publications**	**Total citations**	**Mean citations**	**IF**	**JCR**
Neuroimage	31	15,956	514.7	5.7	Q1
Journal of Applied Physiology	4	1,611	402.8	3.3	Q2
Applied Optics	3	1725	575.0	1.9	Q3
Journal of Biomedical Optics	3	1,096	365.3	3.5	Q2
Journal of Cerebral Blood Flow and Metabolism	3	1,331	443.7	6.3	Q1
Pediatric Research	3	1,338	446.0	3.6	Q1
Physics in Medicine and Biology	3	1,652	550.7	3.5	Q2
Physiological Reviews	3	940	313.3	33.6	Q1
Stroke	3	795	265.0	8.3	Q1
In addition to renaming of some journal, we have used the original journal name in the Results section and reported the IF for 2023.					

### 4.3 Authors, institutes, and countries/regions

**Figure 3:**
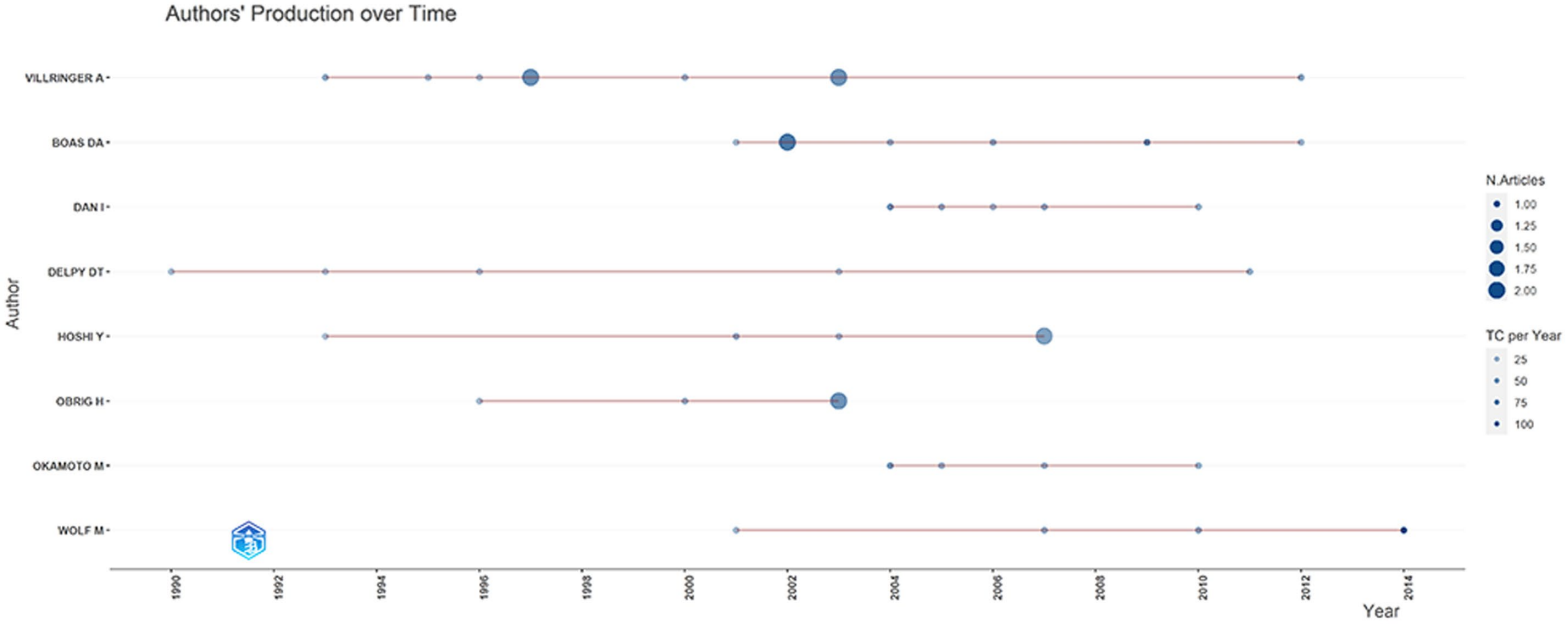
Top 10 authors’ annual outputs between 1990 and 2014.

**Figure 4:**
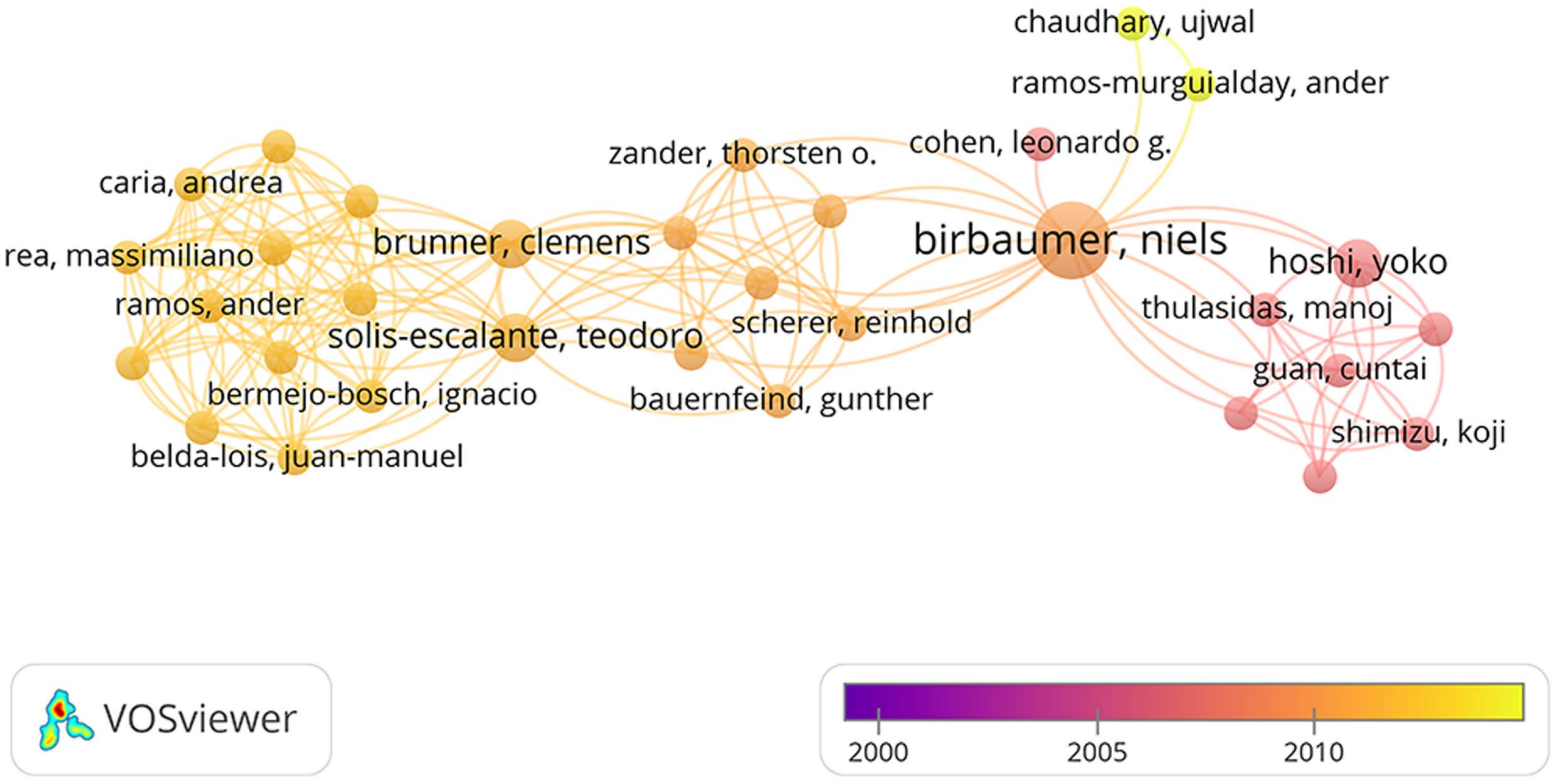
The collaboration between the authors.

Table 2 shows the top 8 authors who published the most top papers. The author with the most published articles is Villringer, A, from Leipzig University. He published nine articles with 5,214 total citations. In this discipline, he is a very significant researcher and his research focuses on the imaging principles of NIRS and standard statistical methods for optical imaging data. With seven articles and 3,551 citations, Boas Da from Harvard University came in second, and his research contents included the application of NIRS and NIRS related head surface relative positioning systems. Dan I from the Nagoya University, Delpy Dt from University College London, and Hoshi Y from Hamamatsu University ranked third. Meanwhile, BOAS DA topped the list in mean citation per article with 638.3. The top authors were mainly from the Japan (*n* = 3), and Germany (*n* = 2). Figure 3 depicts the annual outputs of these top 8 authors between 1990 and 2014. The highly cited articles were published in 1997, 2002, 2003, and 2007 by Villringer, A., Boas, D. A., Hoshi, Y., and Obrig H. Figure 4 shows the collaboration between the authors. Of these, Birbaumer Niels from Tubingen University, Brunner Clemens from Graz University, Teodoro Solis-Escalante from Graz University of Technology and Hoshi Yoko from Hamamatsu University School of Medicine formed the key intermediaries in the authors’ collaborative network.

**Table 2:** Top 8 authors in the top-100 articles.

**Authors**	**Publications**	**Country**	**Total citation**	**Mean citations**	**Institution**
Villringer, A.	9	Germany	5,214	579.3	Leipzig University
BOAS, D. A.	7	USA	4,468	638.3	Harvard University
Dan, I.	5	Japan	2,568	513.6	Nagoya University
DELPY, D. T.	5	England	1709	341.8	University College London
Hoshi, Y.	5	Japan	2020	404.0	Hamamatsu University
Obrig, H.	4	Germany	1979	494.8	Leipzig University
Okamoto, M.	4	Japan	2,265	566.3	University of Tokyo
Wolf, M.	4	Switzerland	2,340	585.0	University of Surrey

**Figure 5:**
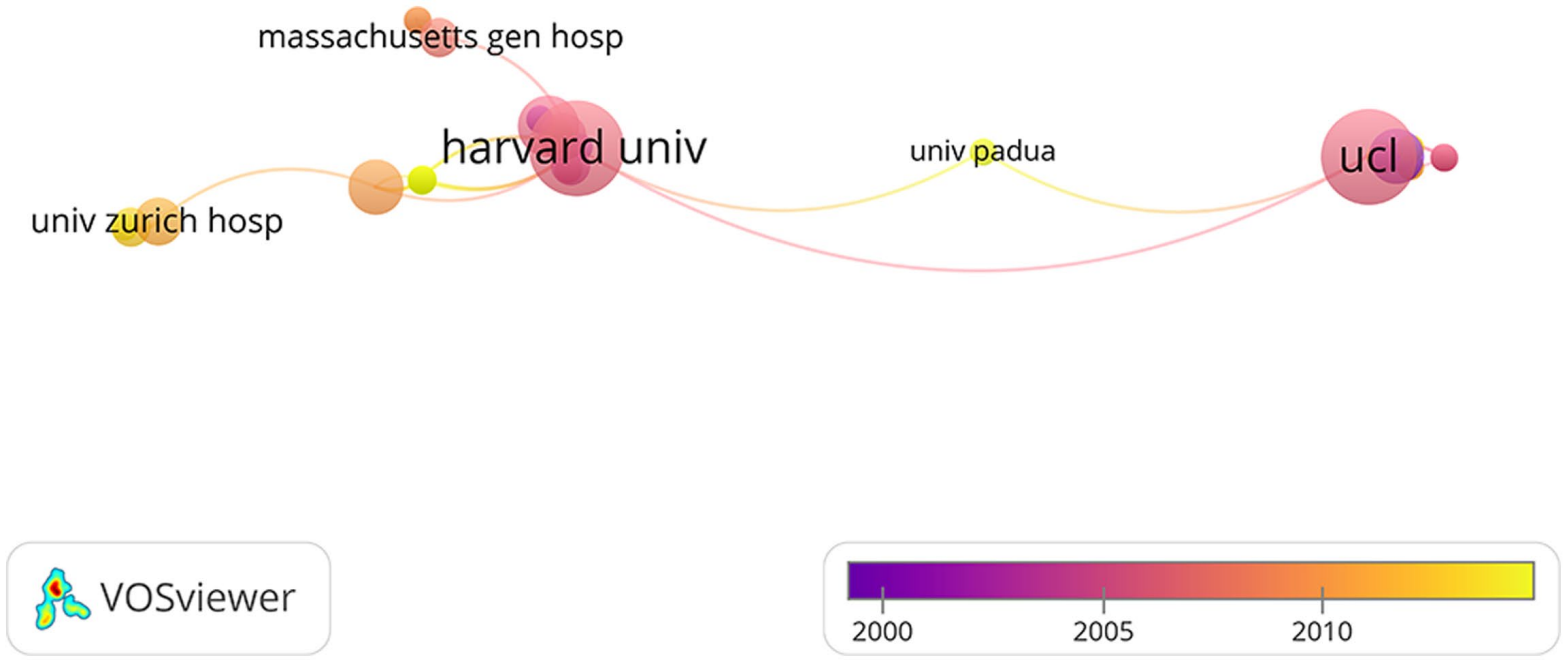
The network visualization between the institutions.

In addition, we analyzed the top 10 institutions that published these 100 articles which is shown in Table 3. The top 10 organizations are mainly from the United States. Harvard University is the top 1 with 22 articles. Followed by National Food Research Center Japan, Stanford University, University Copenhagen, University Tubingen and Washington University tied for second place with 9 articles. The Collaboration Network between the institutions is shown in Figure 5. The university that works most closely with other colleges and universities is Harvard University. Among the institutions that collaborate with Harvard University, University College London publish the higher number of articles. In general, there is close cooperation between University Hospital Zurich, Harvard University, University College London, University of Padua and Massachusetts General Hospital.

**Table 3:** Top 10 institutions in the top-100 articles.

**Institutions**	**Publications**	**Country**
Harvard University	22	USA
National Food Research Center Japan	9	Japan
Stanford University	9	USA
University Copenhagen	9	Denmark
University Tubingen	9	Germany
Washington University	9	USA
Drexel University	7	USA
Humboldt University	7	Germany
Boson Childrens Hospital	6	USA
Tokyo Institute of Psychiatry	6	Japan
University Aquila	6	Italy
University College London	6	US
University Zurich Hospital	6	Switzerland

**Figure 6:**
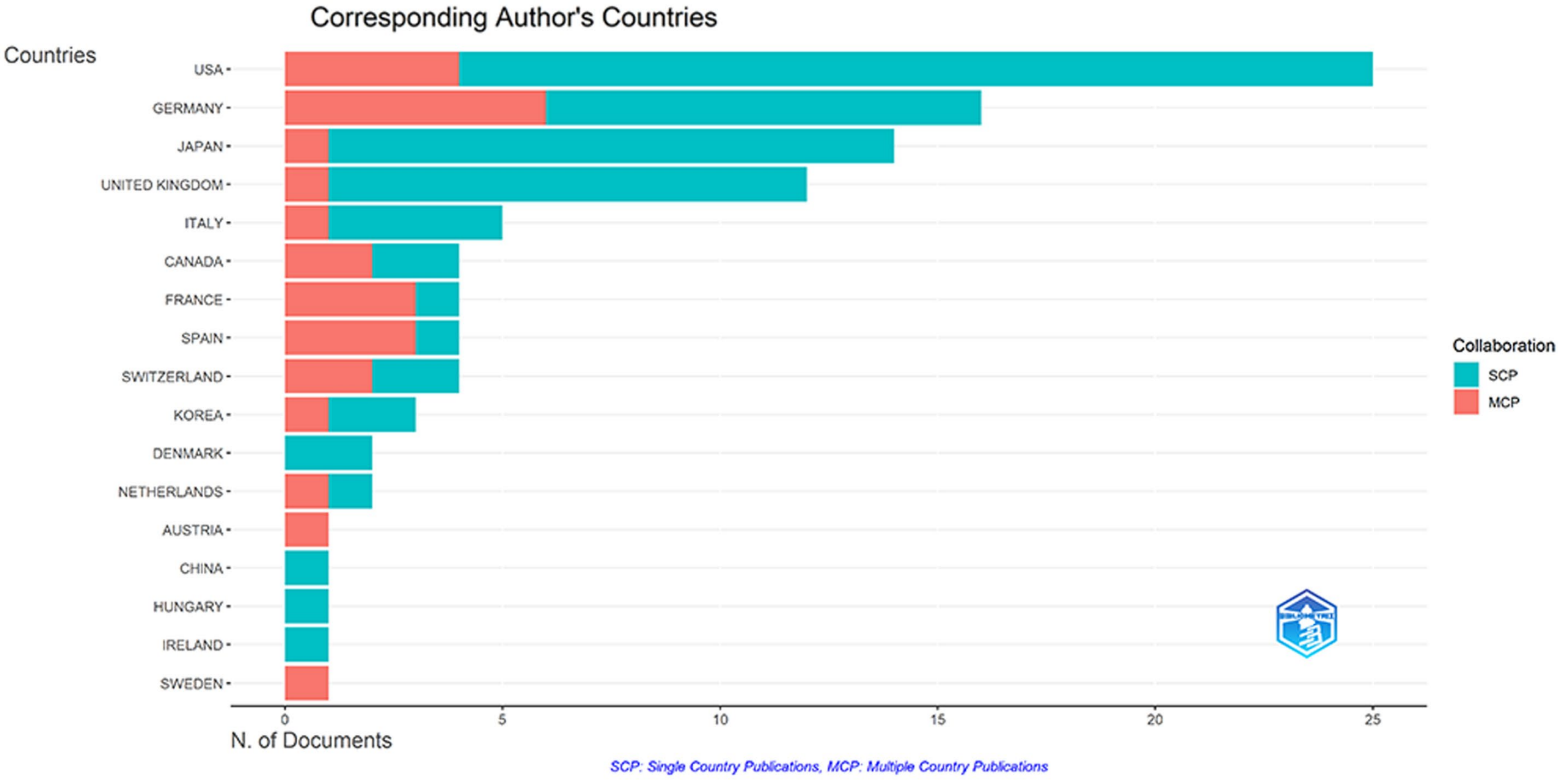
Analysis of corresponding author’s countries.

**Figure 7:**
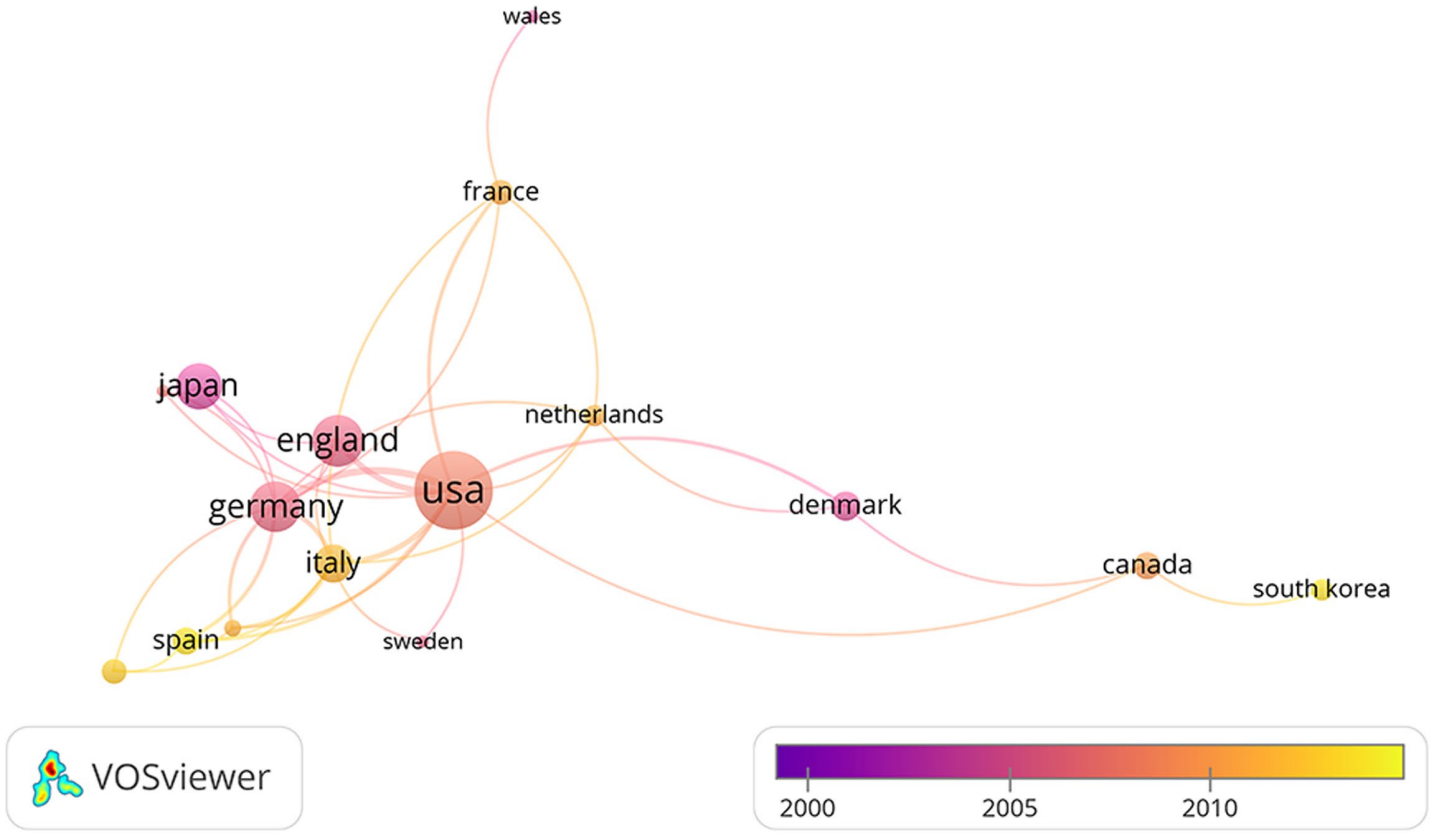
The network visualization between countries.

Overall, 18 countries took part in the top 100 articles in this field. Table 4 shows the number of publications in the top ten countries for the top 100 articles. As the table shows, the United States is in the lead with 11,491 citations, followed by Germany, Japan, and the United Kingdom. Surprisingly, Spain, the sixth with total 2,676 citations, leads the list in terms of average citations with 669.0 citations per article. As shown in Figure 6, the United States was the most productive country, with 25 high-cited articles, of which 4 were multi-country authors (MCP) and 21 were single-country authors (SCP); the followed were Germany (MCP 6+ SCP 10), Japan (MCP 3+ SCP 7), Japan (MCP 0+ SCP 9), France (MCP 3+ SCP 2), Spain (MCP 1+ SCP 13), and the United Kingdom (MCP 1+ SCP 11). Furthermore, the network visualization of the co-authorship analysis between countries was performed in Figure 7. Most international cooperation in the field of highly cited articles is among the United States, Germany, Japan and England. At the same time, Asian countries such as China, Japan, and South Korea have little cooperation.

**Table 4:** Top 10 countries in the top-100 articles.

**Countries/Regions**	**Total citations**	**Average article citations**	**Articles**	**SCP**	**MCP**
USA	11,491	459.6	25	21	4
Germany	7,881	492.6	16	10	6
Japan	6,228	444.9	14	13	1
United Kingdom	4,979	414.9	12	11	1
Italy	3,097	619.4	5	4	1
Spain	2,676	669.0	4	1	3
Switzerland	2,320	580.0	4	2	2
France	1804	451.0	4	1	3
Korea	1,383	461.0	3	2	1
Canada	1,289	322.3	4	2	2

### 4.4 Keywords

**Figure 8:**
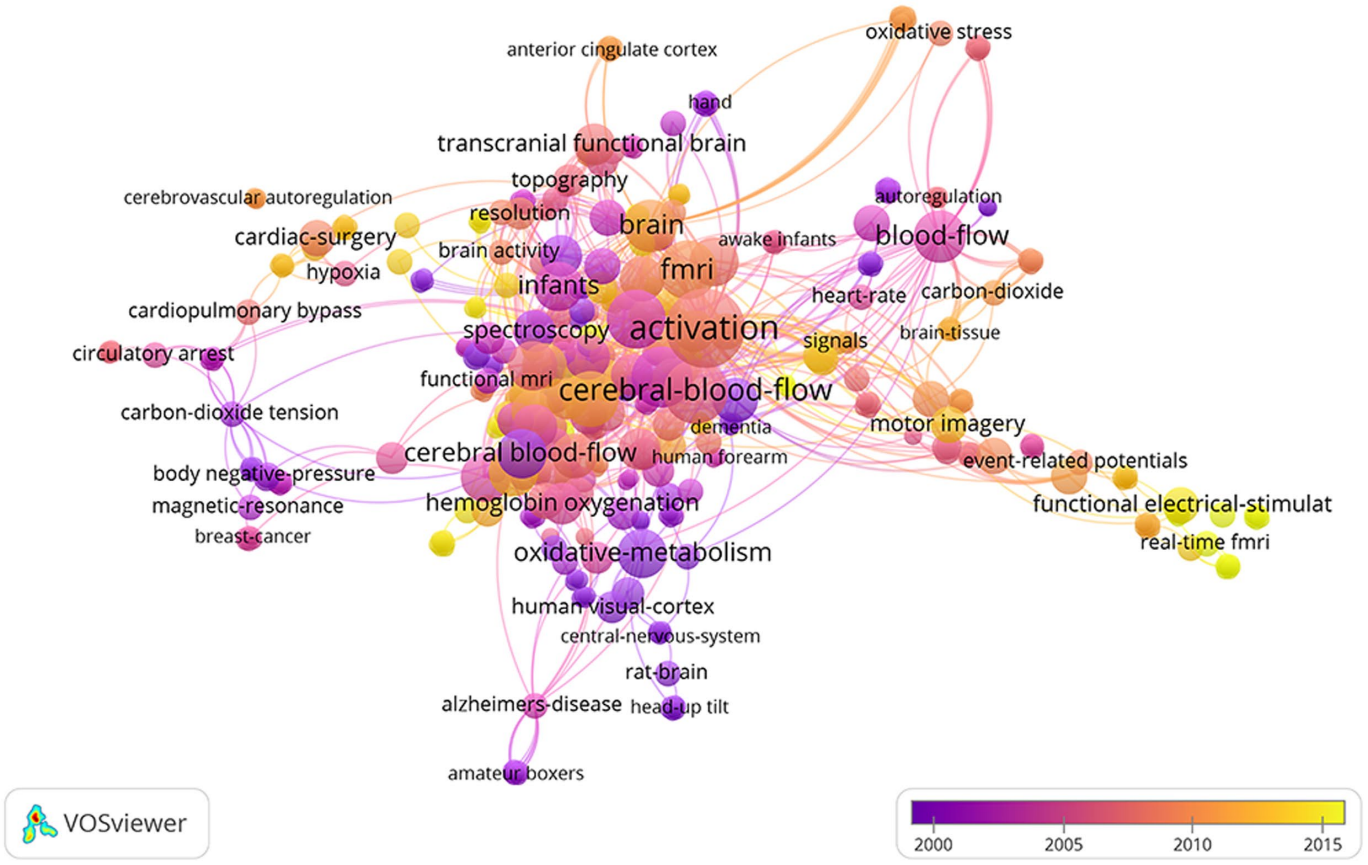
Co-occurrence map of keywords.

Table 5 lists the top 10 keywords and their frequency (four are tied for seventh place). Near-infrared spectroscopy, activation, cerebral-blood-flow, brain, newborn-infants, oxygenation, cortex, fMRI, spectroscopy, stimulation are top 10 keywords. The top keywords with the highest co-occurrence frequency including activation, brain activation, blood oxygenation changes, adsorption, blood-flow, newborn-infants, motor imagery, functional electrical-stimulate, transcranial magnetic stimulate (Figure 8).

**Table 5:** Top-10 keywords in the top-100 articles.

**Keywords**	**Frequency**
Near-infrared spectroscopy	40
Activation	21
Cerebral-blood-flow	12
Brain	11
Newborn-infants	11
Oxygenation	11
Cortex	10
Fmri	10
Spectroscopy	10
Stimulation	10

### 4.5 Funding

A total of 97 institutions funded the 100 articles, with the top eight funds shown in Table 6. National Institutes of Health USA (NIH) and United States Department of Health Human Services tied for first place (n = 28). About 58%of the top funds are from the USA, while the rest are from the United Kingdom, Germany and Swiss. They are almost government agencies.

**Table 6:** The top 8 funds.

**Funding agencies**	**Record count**
National Institutes of Health USA (NIH)	28
United States Department of Health Human Services	28
Nih National Institute of Neurological Disorders Stroke (NINDS)	11
Nih National Center For Research Resources (NCRR)	7
Wellcome Trust	6
Medical Research Council Uk (MRC)	5
Uk Research Innovation (UKRI)	5
Nih National Institute of Biomedical Imaging Bioengineering (NIBIB)	4
German Research Foundation (DFG)	3
Nih Eunice Kennedy Shriver National Institute of Child Health Human Development (NICHD)	3
Stanford Institute For Neuro Innovation And Translational Neurosciences (SINTN)	3
Swiss National Science Foundation (SNSF)	3

## 5 Discussion

In this study, we identified the 100 most cited articles in the field of fNIRS and analyzed their authors, countries, fundings, and keywords. Our analysis found that the top author, institution and country with most top articles were Villringer, A. from University of Pennsylvania, Harvard University and the United States. The relevant important keywords include oxidative-metabolism, functional electrical-stimulation, cardiac-surgery, human visual-cortex, newborn-infants, cardiopulmonary bypass, oxidative stress, fMRI. The National Institutes of Health (NIH USA) supported the most studies among the 100 articles.

Devezas M. Â. M. and Wangwang Yan et al. analyzed the top authors, journals, institutions, countries, and co-occurring keywords network in the field of fNIR research from 2000 to 2020 using different bibliometric indicators and bibliometric analysis tools (30, 41). Xiangyin Ye et al. revealed the overview, hot spots and trends of fNIRS-based clinical disease research from 2011 to 2022; they found that cutting-edge topics include executive function, functional connectivity, Alzheimer’s disease, gait research, etc. (42). And our focus of this research is primarily on analyzing the 100 highly cited articles to identify the authors, institutions, nations, etc. of the highly cited fNIRS articles. This result will aid researchers in understanding the foundation of the fNIRS, identifying the research topic, and identifying fresh viewpoints on the frontier of the field.

When NIRS revealed functional activation (owing to oxygenation and hemodynamic changes) in the human cerebral cortex in 1992 (43), functional mapping of the human brain took on a new level. fNIRS is used to study cortical activity in healthy persons, patients with all kinds of disease, and experimental animals (44, 45). Its use has become more innovative in recent years. Nowadays, its applications include studying the effects of climbing, flying an airplane, and changing sleep states on cerebral hemodynamics, as well as cognitive neuroscience studies with healthy people and patients with various diseases (46).

The most cited was a review published in 2012. The NIR’s contributing historical events are briefly summarized in this article (until 2012) (47). This review emphasizes the technical advancement of fNIRS, including the launch of commercial multichannel systems, commercial wireless instruments in 2012, and more sophisticated prototypes (i.e., from single-point to multipoint functional cortex measurement). It is crucial for the later detection of intracranial hemodynamic response and brain activity in patients with neurological and psychiatric diseases using FNIRS (48, 49). The most cited original article is “Non-invasive optical spectroscopy and imaging of human brain function” published by Villringer (50). And it was published in *Trends in Neurosciences* in 1997. This study introduced that it is already possible to noninvasively measure brain activity in human beings via complete skulls using near-infrared light, which can efficiently permeate biological tissues. It was a major technological breakthrough in brain imaging of NIRS. And this article expounded that NIRS has the advantages of biochemical specificity, millisecond-scale temporal resolution, the capacity to assess intracellular and intravascular events, and the portability of a device suitable for bedside examinations. For the last 5 years, the most cited article was published in *The Annals of The New York Academy of Sciences* called “*the present and future use of functional near-infrared spectroscopy (fNIRS) for cognitive neuroscience”* (24). This review provided a thorough and advanced overview of fNIRS fundamentals, technical development, and application. The potential of fNIRS in cognitive neuroscience research and some unresolved issues were discussed.

The most prolific is Villringer, A. of Leipzig University, who has published nine of the top 100 most cited articles, with a total of 5,214 citations. He then developed the idea that using near-infrared light, which penetrates biological tissues fairly well, could potentially noninvasively assess brain activity in human subjects through an intact skull. He also noted the advantages of the optical approach, including biochemical specificity, temporal resolution in the millisecond range, the potential to measure both intracellular and intravascular events simultaneously, and the portability of devices capable of conducting bedside examinations (50). BOAS DA of Harvard University in the United States ranked second among cited authors, with seven published papers and 4,468 citations. He worked at Massachusetts Hospital, Harvard Medical School as the department’s chief of radiology. He had a hand in five out of the twenty-eight Harvard University articles that received high citation counts. A third-place author with five papers and 2,568 citations was DAN I from Nagoya University. He proposed a method for probabilistic registration of fNIRS data through the international 10–20 system, which was performed through the global 10–20 system to the standard Montreal Neurological Institute (MNI) template without the use of MRI of subjects (51). He demonstrated their method with a new statistical model in the article, making group studies more accessible and enlightening people about the various sources of variability. This study not only improves the reliability of the fNIRS study’s population analysis but will also make it easier for the neuroimaging community to share intramodality and multimodal data. It represents a fresh fNIRS innovation. He also used fNIRS to study the relationship between prefrontal activation and different brain dysfunctions (52–54).

Our analysis showed that the United States had the most high-cited publications, followed by Germany and Japan. Consistent with the results of previous bibliometric analysis, the United States ranks first in the comprehensive influence in this field (30). Almost half of the top 100 articles have been published in these three nations. China ranked third in the total number of articles published in the fNIRS field (1,240 of 9,424 articles, 13.2%), however, research published by China did not enter the top 100 most cited. China’s scientific research, including on fNIRS, has achieved rapid development in the past 10 years. But in terms of cited data, the research done in China needs to further increase its impact. This indicates that China still needs to continue to deepen research in this field.

The top 10 institutions are all from developed countries, which may be related to their national strength and the proportion of investment in scientific research. The top organizations that publish the most top publications are Harvard University, National Food Research Center Japan, Stanford University, University Copenhagen, University Tubingen and Washington University. These six organizations account for 67% of the most cited articles. This demonstrates that in the area of fNIRS, the United States, Japan, Denmark and Germany may lead the globe.

Journal analysis can help researchers to select journals in related fields, understand the journal distribution of publications, and evaluate the overall quality of papers. Most of the articles were published in the journal *Neuroimage*. Some studies were also published in prestigious journals like *Journal of Applied Physiology* and *Applied Optics*. It is worth noting that *Applied Optics,* which is the number three, is a journal in the field of optics. Various compounds have different photophysical characteristics, like brain tissue, which may help to explain why chemistry-related journals appear in our results (55). High IF and JCR journals typically have a substantial academic impact and draw in more high-caliber submissions. We found that the quality of the journals that published these articles varied from Q1 to Q3. These 100 publications have appeared in numerous journals, including medical and optical periodicals. 56 of the 100 articles were published in the top 10 cited journals, demonstrating the importance of these 10 journals in the fNIRS area.

The most extensive collaboration is that which exists between Harvard University and other organizations. It has collaborated with prestigious universities like University College London and University Zurich Hospital. Most of the institutions that Harvard works with are domestic. Intercontinental co-operation is rare. Bibliometrics by productions show that the University of Tubingen, University College London and Beijing Normal University work closely with other institutions (30).

Co-occurrence analysis reveals that keywords is highly correlated with oxidative-metabolism, functional electrical-stimulation, cardiac-surgery, human visual-cortex, newborn-infants, cardiopulmonary bypass, oxidative stress, fMRI. These keywords partially coincide with the burst keywords found by bibliometrics on fNIRS in 2019 (30). As a non-invasive neuroimaging technique, fNIRS tracks variations in cerebral tissue oxygenation in relation to neural activity. The oxygenation of cerebral tissue is continuously monitored, and measurements are made of the alterations in the brain tissue’s optical absorption characteristics. Because of this feature, it can be used to monitor the prediction and evaluation of the treatment efficacy of neurological and psychiatric disorders. As a technique that can detect changes in cerebral cortical blood flow, Yamazaki, R et al. found that the laterality index calculated using fNIRS data may predict the outcome of repetitive TMS (rTMS) in major depressive disorder (MDD) patients (56). It can also be used to comprehensively evaluate the efficacy of some emerging rehabilitation treatment techniques. For example, it can be used not only to monitor the efficacy of repetitive transcranial magnetic stimulation (rTMS) in patients with major depression but also to monitor the response to rTMS in patients with persistent post-concussion symptoms (PPCS) (57). They observed a hemodynamic response induced by the fNIRS task after the working memory task, which showed an increase in oxygenated hemoglobin. In addition, it can be used as a non-invasive instrument to track various cortical activations in the brain as a task is performed. Sun et al. conducted a randomized, double-blind experiment using fNIRS to monitor changes in cortical activation after rTMS treatment of neuropathic pain following spinal cord injury (58). They found that the analgesic mechanism of rTMS may be related to the inhibition of activation of the primary motor cortex (M1) and the premotor cortex (PMC). At the same time, cerebral blood, blood-flow also appear in keyword co-occurrence analysis, which suggests that fNIRS is often used to detect changes in cerebral blood flow. Some studies have used the imaging characteristics of fNIRS to study the cerebral blood flow changes after brain computer interface (BCI) therapy. Further study has revealed that the hybrid fNIRS-EEG-BCI framework can be applied in brain therapy (59). They found that the highest band powers of EEG signals combined with the signal peak and mean fNIRS signals show promise for hybrid BCI. The three frameworks’ combination exhibits significant promise (60) and may develop into a popular research area in the future. In addition to this, fNIRS may be sensitive to physiological alterations during rTMS treatment for neurological and psychiatric conditions. At the same time, this also suggests that research have been done to examine changes in cerebral blood flow following brain computer interface therapy using the imaging capabilities of fNIRS. Concentrates on lately have tracked down that an imaginative mix of close infrared spectroscopy and different FIO2 regimens has shown to be a harmless and dependable method for evaluating cerebrum death. Successful endeavors at NIR spectroscopy have added to the quick and precise evaluation of mind demise, the ideal arrangement of contributor organs with quality confirmation, and a convention help technique for the expansion of NIR spectroscopy applications (3). Likewise, the investigation discovered that NIR spectroscopy can likewise quantify concentration of oxygen and deoxygenated hemoglobin [Δ(HbO2) and Δ(Hb)], diagnose the deep venous thrombosis (DVT), and serve as one of the non-invasive means for post-treatment follow-up. The capability of NIRS for painless, persistent, and direct observing/remedial viability assessment of DVT in a medical clinic or center with fitting bedside checking capacities was identified (4). Alternatively, based on Monte Carlo simulations that visualize light propagation in the head in detail, a study utilizing a noticeable Chinese human dataset that realistically represents human anatomy Hairstyle spatial sensitivity distributions have strong distortions along the folded brain surface. Sensitive brain regions cover the gray matter and extend into the superficial white matter, resulting in a greater depth of penetration (> 3 cm). The study proposes limiting the ideal source-locator separating to 3–3.5 cm, and these outcomes recommend that the cerebral cortex collapsing calculation really generally affects light engendering and is a point that must be considered in the application of functional NIR spectroscopy (61). For more than 20 years, fNIRS has enabled clinicians to understand the mechanisms of brain development and damage in newborns and is a useful addition to existing technology. fNIRS applications in neonates fall into two distinct categories: task-related studies and hemoglobin phase transition studies. Identifying normal, healthy haemodynamic responses in newborns using fNIRS will help identify unhealthy patterns and their association with normal neurodevelopment (62). However, when used in newborns, its monitoring depth and spatial resolution are limited (15).

## 6 Limitations

There are several limitations to our study. First of all, Scopus, Web of Science, and PubMed databases are the three mainstream databases in medicine. Research has found that Scopus covers a broader range of journals and is helpful in keyword searches and citation analysis. Still, it is currently limited to recent articles (published after 1995) compared to Web of Science (63). We chose Web of Science as the search engine and may have missed some articles only included in Soups. Secondly, we cannot assess certain recently released high-cited studies because the most commonly cited articles have been around for a while (64). Furthermore, not all of the articles may have been covered by the keywords employed in this analysis. This is a drawback of bibliometrics (65). Additionally, because of the time lag between the article’s publication and the Web of Science collection, the article’s citation times vary over time. It is necessary to update for future studies in order to comprehend the most recent research foundations in the field of fNIRS. In the future, expanding the scope of the search database and increasing the number of search databases may reduce this limitation.

## 7 Conclusion

We have compiled a list of the 100 most cited articles in the fNIRS field to identify current status and global trends. According to our bibliometric research, the United States continues to lead the globe in this area. Villringer A. and Harvard University were the most productive author and institution for highly cited articles. The countries with the highest number of articles are the United States. Articles from Germany, Japan and Spain were the next most cited. The scholarly journals with the most impact in this area include *Neuroimage*, *Journal of Biomedical Optics,* and *Physiological Reviews*. Activation, blood oxygenation changes, adsorption, blood-flow, newborn-infants, motor imagery, functional electrical-stimulate, TMS, oxygenation, fMRI, hemodynamic-response, diffuse optical tomography were the hotspots for fNIR. These results assist the researcher in better grasping the standard and trend of fNIRS research and how to use the canonical articles. We appeal to the relevant researchers of fNIRS, hoping to strengthen the research of fNIRS in the field of technology and treatment according to our research results, and communicate to promote the development and application of this technology.

## Data availability statement

The datasets presented in this study can be found in online repositories. The names of the repository/repositories and accession number(s) can be found in the article/supplementary material.

## Ethics statement

The study was exempted from institutional ethics review because only publicly available data were analyzed.

## Author contributions

JL: Writing – review & editing, Data curation, Software, Writing – original draft. YL: Writing – original draft, Supervision. MH: Writing – original draft, Formal analysis. DL: Writing – original draft, Resources. TW: Writing – original draft, Project administration. FS: Writing – original draft. QZ: Writing – original draft. FX: Conceptualization, Methodology, Writing – review & editing. JW: Writing – review & editing, Funding acquisition.
